# Hyperplastic Cardiac Sarcoma Recurrence

**DOI:** 10.1155/2015/132328

**Published:** 2015-02-15

**Authors:** Masood A. Shariff, Juan A. Abreu, Farida Durrani, Eddie Daniele, Kimberly C. Bowman, Scott Sadel, Kourosh T. Asgarian, Joseph T. McGinn, John P. Nabagiez

**Affiliations:** ^1^Department of Cardiothoracic Surgery, Staten Island University Hospital, 475 Seaview Avenue, Staten Island, NY 10305, USA; ^2^Department of Surgery, Staten Island University Hospital, 475 Seaview Avenue, Staten Island, NY 10305, USA; ^3^Department of Pathology, Staten Island University Hospital, 475 Seaview Avenue, Staten Island, NY 10305, USA; ^4^Department of Cardiac Anesthesiology, Staten Island University Hospital, 475 Seaview Avenue, Staten Island, NY 10305, USA

## Abstract

Primary cardiac sarcomas are rare tumors with a median survival of 6–12 months. Data suggest that an aggressive multidisciplinary approach may improve patient outcome. We present the case of a male who underwent resection of cardiac sarcoma three times from the age of 32 to 34. This report discusses the malignant nature of cardiac sarcoma and the importance of postoperative multidisciplinary care.

## 1. Introduction

Primary cardiac sarcomas comprise less than 1% of sarcomas, 75% being benign and fatal with location [1]. Symptoms include arrhythmias and dyspnea due to pulmonary hypertension [2]. Delay in diagnosis occurs because symptoms do not manifest until the mass is large enough to impede blood flow. Resection is followed by monitoring for recurrence [3]. Failure to completely remove the tumor results in recurrence within 6–12 months. Postoperative treatment is dictated by morphology, location, and immunohistochemistries. 75% occur within the left atrium, a positive prognostic factor [4]. We report a young patient who underwent resection and two repeat resections for two recurrences.

## 2. Presentation 1

In March of 2009, a 32-year-old male presented with shortness of breath and chest pain. Cardiac enzymes were negative and a CT scan revealed a large left atrial mass. A transthoracic echocardiogram (TTE) confirmed the left atrial mass with severely hypoechoic perimeters attached to the posterior aspect of the left atrium. The patient was hemodynamically unstable and complete resection was performed via sternotomy with transseptal exposure of the left atrium. The tumor was attached to the free wall of the left atrium and the undersurface of the mitral valve. The entire epicardium was excised with the tumor over the left atrium, exposing the muscle of the left atrium to the inner atrium. Pathology revealed a 5.7 × 4.7 cm high grade sarcoma with evidence of muscle differentiation and immunohistochemical studies revealed strongly positive vimentin. The patient was discharged on postoperative day seven with instructions to follow up with the surgeon and an oncologist.

## 3. Presentation 2

**Figure 1:**
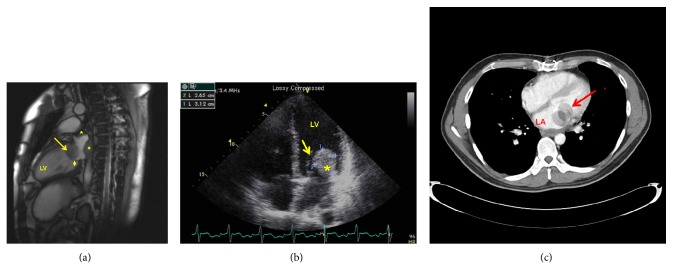
Presentation 2. (a) MRI showing mass in the left side of the heart pressing on the mitral valve (arrow head) and two other masses in the left atrium indicated (asterisks). (b) TEE showing a mobile, echogenic mass (asterisk) in the left heart prolapsing through the mitral valve (arrow) into the ventricle. (c) Nongated CT-scan captured the mass (arrow) in motion in the left side of the heart. LA: left atrium. LV: left ventricle.

In September of 2009 the patient presented with shortness of breath, chest pain, and palpitations. He had not followed up with an oncologist due to financial hardship. CT and MRI scans revealed three lobular enhancing masses within the left atrium. The largest, measuring 3.5 × 2.6 cm, was attached to the lateral wall of the atrium and on the posterolateral leaflet of the mitral valve, protruding into the left ventricle with extension into the left inferior pulmonary vein. The second mass was broad based posteriorly near the origin of the left inferior pulmonary vein. The third mass was in the interatrial septum (Figure 1). Liver and pulmonary metastases were noted. TTE revealed the mass causing 2+ mitral regurgitation with normal valvular morphology and an ejection fraction of 50% (Figure 1).

**Figure 2:**
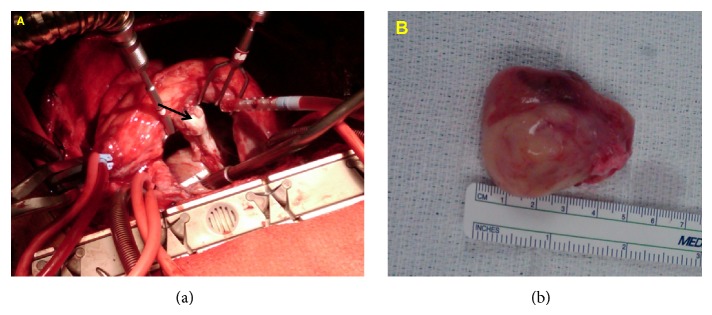
Presentation 2. (a) Tumor in the posterior aspect of the left atrium (arrow). (b) Excised left atrial sarcoma.

The patient underwent a redo-sternotomy with cardiopulmonary bypass. A transseptal en bloc resection was performed including the posterior aspect of the pericardium, the entire left atrium up to the left pulmonary veins, and 1 cm from the annulus of the mitral valve (Figure 2). The mitral valve, at gross examination, did not have any involvement/infiltration of the tumor contrary to the TTE and radiologic imaging and was left intact. The right atrium was then resected up to the atrial septum into the coronary sinus at the junction of the superior and inferior vena cavae. Additional tumor extension along the pericardium was also excised. The posterior left atrium was reconstructed using bovine pericardial patch, including pulmonary vein inflow. Bovine patch was utilized to reapproximate the right atrium anteriorly.

**Figure 3:**
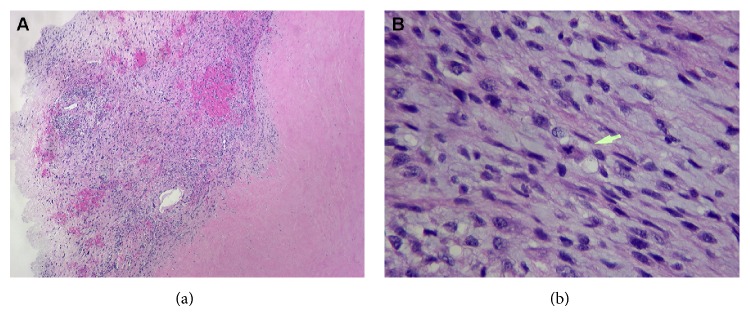
(a) Cellular neoplasm with infiltrative borders and necrosis (×10). (b) The high power (×40) reveals a cellular tumor with high nuclear/cytoplasmic ratio, irregular hyperchromatic, pleomorphic nuclei exhibiting high mitotic activity with abnormal mitosis and giant cells (arrow). Digital images captured with a digital camera (Olympus dp 20) attached to a microscope (Olympus b × 51).

Pathology revealed malignant fibrous histiocytoma, lobulated and invasive into the myocardial tissue, with mitosis band, signifying the differentiation of the tumors, and strongly positive vimentin and CD68 stain (Figure 3). The patient was discharged on postoperative day five on warfarin with oncology follow-up. Adjuvant chemotherapy was administered with resolution of nodules in the liver and lung. MRI in January 2010 and CT scan in December showed no evidence of regrowth. The morphology and architecture of the atria were well defined without any filling defects.

## 4. Presentation 3

**Figure 4:**
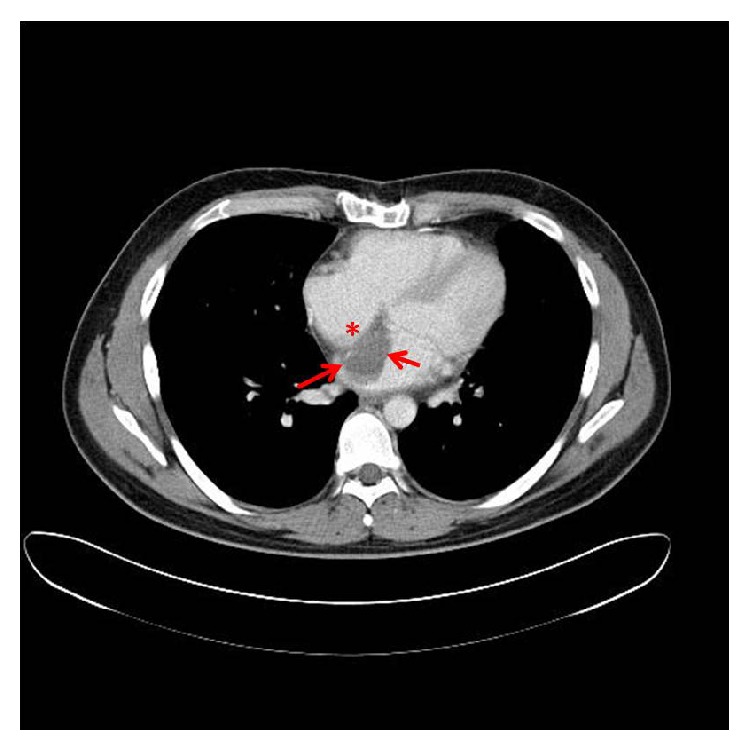
Presentation 3. CT-scan showing tumor of the intra-atrial septum in the left atrium, nongated scan.

A CT scan in March of 2011 revealed a 2.8 × 3.7 cm left atrial recurrence 10 weeks after completion of chemotherapy. TTE revealed an echogenic density in the left atrium originating in the interatrial septum (Figure 4). Redo-sternotomy was performed with cardiopulmonary bypass. Tumor was resected from the interatrial septum in the vicinity of the previously placed patch, including the left superior and inferior pulmonary veins. Resection continued over the anterior leaflet of the mitral valve annulus. Pathology confirmed negative margins inferior to the interatrial septum, but microscopic disease was noted on the posterior mass adjacent to the mitral valve leaflet connecting to the ostium of the pulmonary veins. Resection of the latter was impossible without resecting the entire fibrous trigone of the heart and possible left pneumonectomy. The interatrial septum and the free wall of the left and the right atrium were reconstructed with bovine pericardium. Pathology revealed a high-grade spindle cell and pleomorphic sarcoma. The patient was discharged on postoperative day six.

The patient received adjuvant chemotherapy. The follow-up scans were negative for regrowth in the heart, but the tumor had spread in the pulmonary cavity with extrathoracic manifestations in the brain and liver. Chemotherapy and radiation (Gamma Knife radiosurgery) were utilized in 2012, with limited regrowth and partial response. Early 2013, the patient was admitted into acute care there upon progressed to multiorgan failure and expired on March 2013. The patient's survival after primary resection was 48 months.

## 5. Comments

This case demonstrates the aggressive nature of cardiac sarcoma. The completeness of resection is considered a primary determinant to long-term survival. The goal is to achieve an R0 resection with adequate margins if possible when there is no evidence of extracardiac disease, or surgical debulking if deemed unresectable [4]. Most left sided tumors are first seen with heart failure and require prompt resection followed by adjuvant chemotherapy including doxorubicin, ifosfamide, gemcitabine, and docetaxel [5]. Positive prognostic indicators are left sided location, mitotic rate less than 10 per high power field and absence of necrosis on histology [6]. Postoperative chemotherapy decreases recurrence. Prophylactic resection of this patient's interatrial septum might have improved his prognosis. With adjuvant therapy the patient remained in remission for 18 months.

The malignant nature of these tumors is well established and even after adjuvant therapy mortality is high [3]. Incomplete resection requires chemotherapy and radiation [4, 7]. Palliative radiation therapy is recommended if resection is not feasible [4]. Sarcomas originating in a chamber other than the left atrium demonstrate increased recurrence. Atrial reconstruction with a pericardial patch is feasible to completely resect the tumor whereas ventricular growth and involvement of the valve typically require transplantation [8]. The extracardiac manifestations in our report with brain metastasis demonstrates the need for a multidisciplinary approach to dealing with cardiac sarcomas [4, 7, 9].

This case is notable for the short interval to recurrence: 6 months between the first and second presentation and 4 months between completion of chemotherapy and the diagnosed second recurrence. Completeness of resection at first presentation is a positive feature in disease-free survival. Without adjuvant chemotherapy recurrence of cardiac sarcomas increases, as demonstrated in this case. Recurrence of primary cardiac sarcomas is common and close surveillance with an oncologist is essential.

## Supplementary Material

Video 1: MRI heart morphology at Presentation 2, depicting the mass attached to the lateral wall of the atrium and on the posterolateral leaflet of the mitral valve, prolapsing into the ventricle. The three masses can be seen in the left atrium interfering with blood flow and presentation of symptoms.
Video 2: Echocardiogram at Presentation 2 showing the mobile, echogenic mass in the left heart prolapsing through the mitral valve into the ventricle in various cross sectional views.
